# A genome-wide CRISPR screen identifies DPM1 as a modifier of DPAGT1 deficiency and ER stress

**DOI:** 10.1371/journal.pgen.1010430

**Published:** 2022-09-27

**Authors:** Hans M. Dalton, Raghuvir Viswanatha, Roderick Brathwaite, Jae Sophia Zuno, Alexys R. Berman, Rebekah Rushforth, Stephanie E. Mohr, Norbert Perrimon, Clement Y. Chow

**Affiliations:** 1 Department of Human Genetics, University of Utah School of Medicine, Salt Lake City, Utah, United States of America; 2 Department of Genetics, Blavatnik Institute, Harvard Medical School, Boston, Massachusetts, United States of America; 3 Howard Hughes Medical Institute, Boston, Massachusetts, United States of America; RIKEN Advanced Science Institute, JAPAN

## Abstract

Partial loss-of-function mutations in glycosylation pathways underlie a set of rare diseases called Congenital Disorders of Glycosylation (CDGs). In particular, DPAGT1-CDG is caused by mutations in the gene encoding the first step in N-glycosylation, *DPAGT1*, and this disorder currently lacks effective therapies. To identify potential therapeutic targets for DPAGT1-CDG, we performed CRISPR knockout screens in *Drosophila* cells for genes associated with better survival and glycoprotein levels under DPAGT1 inhibition. We identified hundreds of candidate genes that may be of therapeutic benefit. Intriguingly, inhibition of the mannosyltransferase Dpm1, or its downstream glycosylation pathways, could rescue two *in vivo* models of *DPAGT1* inhibition and ER stress, even though impairment of these pathways alone usually causes CDGs. While both *in vivo* models ostensibly cause cellular stress (through *DPAGT1* inhibition or a misfolded protein), we found a novel difference in fructose metabolism that may indicate glycolysis as a modulator of DPAGT1-CDG. Our results provide new therapeutic targets for DPAGT1-CDG, include the unique finding of *Dpm1*-related pathways rescuing *DPAGT1* inhibition, and reveal a novel interaction between fructose metabolism and ER stress.

## Author summary

Sugar modifications are important to proper protein folding and function. The biological pathways that control these sugar modifications are called glycosylation pathways. When these pathways are disrupted, such as through genetic mutation, it can cause rare developmental disorders called Congenital Disorders of Glycosylation (CDGs). Most CDGs lack any treatment options, and little is known about interacting genes or pathways that might improve these disorders. Here, we study DPAGT1-CDG which involves mutations in the enzyme-encoding gene, *DPAGT1*, by using the fruit fly model organism, *Drosophila melanogaster*. Using inhibitory drugs, we perform two cell-based screens with CRISPR technology to determine which genes are important under DPAGT1 inhibition. We then used a living fly, that models DPAGT1-CDG in the eye, in order to determine which of the top screened genes can improve its impaired eye development. Surprisingly, we found that inhibiting other CDG-causing genes, such as our top hit *Dpm1*, could improve our DPAGT1-CDG cell and fly models. In addition, loss of certain metabolism genes, and changes in diet, also improved the DPAGT1-CDG model. This study finds multiple new genes and pathways implicated in DPAGT1-CDG and lays the groundwork for new CDG therapies.

## Introduction

Glycosylation comprises a variety of sugar-based co- or post-translational modifications that occur primarily in the endoplasmic reticulum (ER) and Golgi apparatus [1]. It includes N-, O-, C-, and S-linked bonds as well as C-terminal-linked GPI anchors. Mutations in glycosylation pathways underlie rare diseases known as Congenital Disorders of Glycosylation (CDGs) [2]. CDGs can be caused by defects of enzymatic machinery or in transport of enzymatic substrates (such as UDP-Gal or sialic acid) [3]. CDGs have limited treatment options. While some treatments correct the cause of the disease, such as dietary sugar supplementation, most only alleviate symptoms [3,4]. Research into the mechanisms of these pathways should help provide better avenues for advancing patient care.

Of the glycosylation pathways, N-linked glycosylation is critical for proper protein folding and function [1,5]. Inhibition of or abnormal N-linked glycosylation causes a build-up of misfolded proteins in the ER lumen, ER stress, and induction of the unfolded protein response (UPR) [5–10]. The UPR is a transcriptional response that attempts to restore homeostasis to the cell, but it can also lead to apoptosis when the stress is not resolved. The initial metabolite required for N-glycosylation is GlcNAc-PP-dolichol, which is synthesized by the dolichyl-phosphate N-acetylglucosamine phosphotransferase 1 (DPAGT1) enzyme [11] (*Alg7* in *Drosophila*, hereafter referred to as *DPAGT1*). In humans, partial loss-of-function mutations in *DPAGT1* can cause DPAGT1-CDG (CDG-Ij) with symptoms including seizures and developmental delay, among others [11,12]. Mutations in *DPAGT1* are also associated with congenital myasthenic syndrome due to hypoglycosylated acetylcholine receptors [13,14]. Inhibiting DPAGT1 with the nucleoside Tunicamycin (Tun) reduces GlcNAc-PP-dolichol levels [15,16] and induces ER stress [17]. DPAGT1-CDG patient cells are more sensitive to Tun [12], and *DPAGT1* mutant cell lines also show an increased level of chronic ER stress, autophagy, and have signs of senescence [18].

As with most CDGs, there are only symptomatic treatment options and no causative therapeutics available for DPAGT1-CDG [14,19]. Any therapeutic option must be precise, as overexpression of *DPAGT1* can also lead to improper N-glycosylation and may underlie some cancer phenotypes [20]. One alternative approach to current therapeutics is to identify modifier genes, where targeting genes that interact with *DPAGT1* expression or downstream phenotypes can potentially be used to develop new therapeutic options [21–23]. Moreover, determining if patients have differential baseline expression of these modifier genes may result in better personalized therapeutic solutions [22].

Here we present two genome-wide CRISPR screens for genes that modify phenotypes associated with reduced function of *DPAGT1*. These screens identify novel genes involved in modifying cellular and physiological outcomes associated with DPAGT1 inhibition and increased ER stress. Of note, we find that knockout of multiple GPI anchor biosynthesis genes improves survival and cell surface glycoprotein levels in *Drosophila* S2 cells associated with *DPAGT1* inhibition and ER stress from tunicamycin. In addition, we find that the mannosyltransferase *Dpm1* is one of the strongest modifier genes and inhibition of *Dpm1* vastly improves cell survival under the loss of *DPAGT1* function and ER stress. We provide evidence that this effect likely occurs through the combined role of Dpm1 in O-mannosylation, N-glycosylation, and GPI anchor biosynthesis pathways. Importantly, the majority of our top candidate genes validate in *in vivo* models of *DPAGT1* inhibition and ER stress. In addition, results from the cell screens suggest that fructose metabolism might affect the loss of *DPAGT1* function, and we observed altered fructose metabolism in the *in vivo DPAGT1* model, suggesting that dietary fructose supplementation may hold therapeutic promise for these patients. Taken together, we identify numerous new candidate *DPAGT1* modifier genes, using *Drosophila* flies and cells, that are potential therapeutic targets and report the remarkable finding that impairing CDG genes in parallel can result in an improved overall phenotype.

## Results

### Disruption of glycosylation and glycolytic pathways rescue the effects of *DPAGT1* inhibition

**Fig 1:**
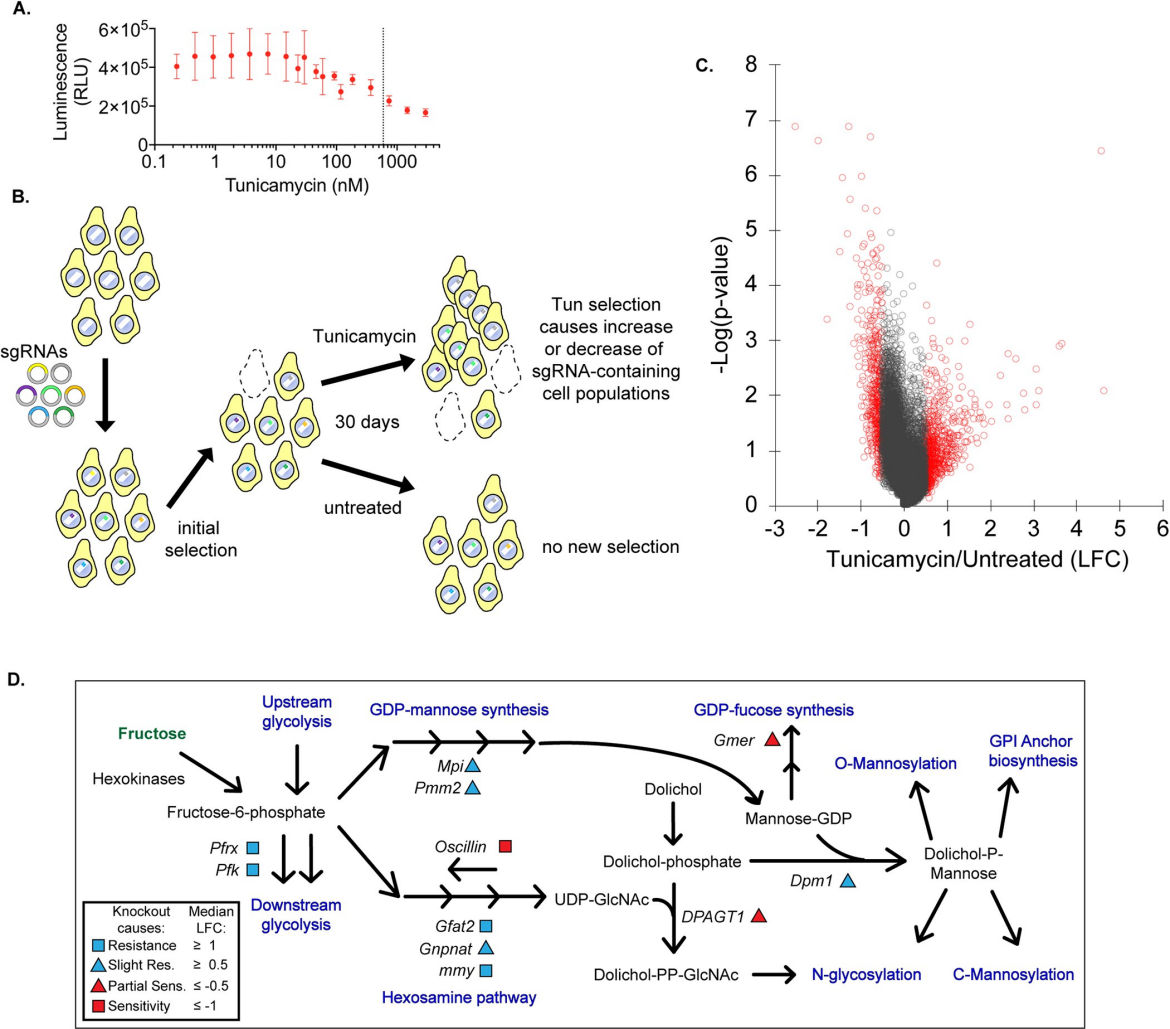
A survival screen reveals hexosamine, glycolysis, and GDP-mannose synthesis in resistance to *DPAGT1* loss. (A) We used a cell titer assay to determine the concentration of Tun capable of reducing cell growth rate by ~50% for use in both follow-up screens. (B) We introduced a whole genome guide RNA library into Drosophila cells stably expressing constitutive Cas9. Pooled cell populations were grown for 30 days either untreated or with Tun. Final cell populations were sequenced for sgRNA abundance to determine candidate genes implicated in Tun resistance or sensitivity. (C) Volcano plot of survival screen. Red dots indicate genes with an absolute lfc value of ≥0.5 or ≤-0.5. (D) A simplified model of one set of highly enriched pathways for gene knockouts causing resistance or sensitivity. Note that only genes whose knockout provided resistance or sensitivity are labeled.

To identify genes impacting the loss of *DPAGT1* function and ER stress, we performed a cell-based, genome-wide CRISPR/Cas9 knockout screen (hereafter referred to as "survival screen"). Briefly, we used genome-wide single guide RNA (sgRNA) libraries transfected into a *Drosophila* S2 cell line to generate a pool of cells in which each cell carried gene knockouts (as described previously [24,25], Fig 1). Each gene tested had at least four different sgRNAs per gene in order to mitigate sgRNA-specific effects. After gene knockout, cells were either untreated or exposed to a sublethal dose of the DPAGT1 inhibitor Tunicamycin (Tun; 500ng/ml [590nM]). This was determined by a titration assay and selection of a dose capable of ~50% reduction in growth rate (Fig 1). After 30 days under DPAGT1 inhibition, cell populations were subjected to deep amplicon sequencing to determine sgRNA abundance. A change in the abundance of an sgRNA in the final population of DPAGT1-inhibited cells vs. untreated cells indicated that knockout of that gene caused resistance or sensitivity. Genes were ranked by the median log-fold change (lfc) in the abundance of each sgRNA targeting that gene (Tables 1 and 2 and S1).

**Table 1:** Summary of survival screen data.

Resistance genes			Sensitivity genes		
lfc	Total hits	% total genes	lfc	Total hits	% total genes
2 or higher	16	0.12%	-2 or lower	1	0.01%
1–1.99	156	1.18%	-1 –-1.99	9	0.07%
0.5–0.99	979	7.40%	-0.5 –-0.99	241	1.82%

**Table 2:** Top 20 candidate genes providing resistance and sensitivity.

Fly Gene	Human ortholog	lfc	Fly Gene	Human ortholog	lfc
*PEK*	*EIF2AK3*	4.76	*pnt*	*ETS1*	-2.41
*Gfat2*	*GFPT2*	4.68	*Oscillin*	*GNPDA2*	-1.87
*CG9609*	*GTF3A*	3.78	*upSET*	*KMT2E*	-1.66
*Hrd3*	*SEL1L*	3.71	*CG9590*	*FAM114A2*	-1.37
*Ide*	*IDE*	3.25	*GalT1*	*B3GALT1*	-1.31
*drongo*	*AGFG1*	3.19	*Asx*	*ASXL3*	-1.18
*CG30345*	*SLC46A1*	3.17	*CG10984*	*ANKRD12*	-1.17
*EcR*	*NR1H3*	2.94	*Vap-33A*	*VAPB*	-1.12
*ham*	*MECOM*	2.91	*Rab40*	*RAB40C*	-1.12
*srp*	*GATA1*	2.72	*Cdk2*	*CDK2*	-1.05
*sau*	*GOLPH3*	2.53	*bel*	*DDX3X*	-0.98
*Pten*	*PTEN*	2.52	*P5cr-2*	*PYCR3*	-0.96
*Sin3A*	*SIN3A*	2.52	*Tango14*	*NUS1*	-0.95
*Cdep*	*FARP2*	2.37	*vtd*	*RAD21*	-0.95
*CG10960*	*SLC2A8*	2.16	*CG33110*	*ELOVL1*	-0.95
*REG*	*PSME3*	2.08	*CG32079*	*SLC36A2*	-0.95
*dl*	*REL*	1.98	*Tor*	*MTOR*	-0.94
*ND-MLRQ*	*NDUFA4*	1.97	*Oatp58Da*	*SLCO1A2*	-0.93
*CG2926*	*PHRF1*	1.94	*dre4*	*SUPT16H*	-0.90
*Gpdh1*	*GPD1*	1.90	*sws*	*PNPLA7*	-0.90

Human orthologs are based on the highest DIOPT score [26].

1151 gene knockouts (8.7% of genes tested) increased resistance to DPAGT1 inhibition at an lfc value of ≥0.5, while 251 gene knockouts (1.9% of genes tested) increased sensitivity to DPAGT1 inhibition at an lfc value of ≤-0.5 (Table 1). Top resistance genes include the major ER stress sensor *PEK* (human: *PERK;* lfc = 4.76), hexosamine enzymes *Gfat2* (*GFPT2*; lfc = 4.68) and *mmy* (*UAP1*; lfc = 1.57), the insulin-degrading enzyme *Ide* (*IDE*; lfc = 3.25), and the glycolytic kinase *Pfk* (*PFKM*; lfc = 1.47) and the kinase/phosphatase *Pfrx* (*PFKFB1*; lfc = 1.74) (Tables 2 and S1). Top sensitivity genes include the proto-oncogene *pnt* (*ETS1*; lfc = -2.41), the hexosamine enzyme *Oscillin* (*GNPDA2*; lfc = -1.87), and the transporter-related genes *Rab6* (*RAB6A*; lfc = -0.85) and *Rab40* (*RAB40C*; lfc = -1.12) (Tables 2 and S1).

Many top candidate genes are *Drosophila* orthologs of known CDG genes. To explore this further, we queried all known *Drosophila* CDG orthologs and a CDG genetic panel [27,28] (145 genes, S2 Table). We considered genes for which knockout increased sensitivity (6 genes) separately from genes for which knockout increased resistance (23 genes). We did not perform gene enrichment analysis for the ‘increased sensitivity’ genes due to lack of statistical power. Remarkably, there was a significant enrichment of CDG genes among genes for which knockout increased resistance to DPAGT1 inhibition (23 observed vs. 12.6 expected, p<0.01, S2 Table). This suggests that perturbation of certain CDG-related pathways in parallel can result in an overall improvement to cellular health.

We performed Gene Ontology (GO) analysis on all candidate genes with an absolute lfc ≥ 0.5 compared to untreated cells (Tables 3 and S3). We chose to use both resistance and sensitivity genes in the same analysis, as many pathways include genes that are either positive or negative regulators. The top enriched categories included "nucleotide-sugar biosynthetic process" (GO:0009226) and "pyruvate metabolic process" (GO:0006090). These include genes involved in the hexosamine pathway and glycolysis, respectively, and relate to *DPAGT1*, as both pathways are critical in synthesizing upstream metabolites for several glycosylation pathways [29]. To further explore these connections, we also examined an adjacent pathway (GO term "GDP-mannose metabolic process", GO:0019673). This pathway involves the creation of GDP-mannose from fructose-6-phosphate, which includes the most common CDG gene *Pmm2* (*PMM2*; lfc = 0.50), and is also important in glycosylation pathways [1]. Here, we found multiple genes at an lfc value cutoff of ±0.5 (4/7 *Drosophila* orthologs, Fig 1C). Overall, there is strong enrichment of genes in glycolytic, hexosamine, and related pathways, suggesting the hypothesis that suppression of these pathways rescues the effects of DPAGT1 inhibition.

**Table 3:** Gene ontology analysis of top candidate genes from the survival screen.

Gene Ontology	Fold Enriched	FDR
nucleotide-sugar biosynthetic process (GO:0009226)	6.57	3.85E-02
nucleotide-sugar metabolic process (GO:0009225)	6.34	5.26E-03
regulation of feeding behavior (GO:0060259)	4.38	4.40E-02
female germ-line stem cell population maintenance (GO:0036099)	3.94	1.11E-02
larval midgut histolysis (GO:0035069)	3.52	4.55E-02
cuticle pattern formation (GO:0035017)	3.39	3.74E-02
midgut development (GO:0007494)	3.38	2.58E-02
pyruvate metabolic process (GO:0006090)	3.37	1.78E-02
cell surface receptor signaling pathway involved in cell-cell signaling (GO:1905114)	3.29	4.42E-02
synaptic assembly at neuromuscular junction (GO:0051124)	3.29	4.40E-02

Candidate genes with an lfc of ≥0.5 or ≤-0.5 were analyzed together.

### Loss of GPI anchor biosynthesis rescues DPAGT1 inhibition-induced reduction in cell surface glycoprotein levels

**Fig 2:**
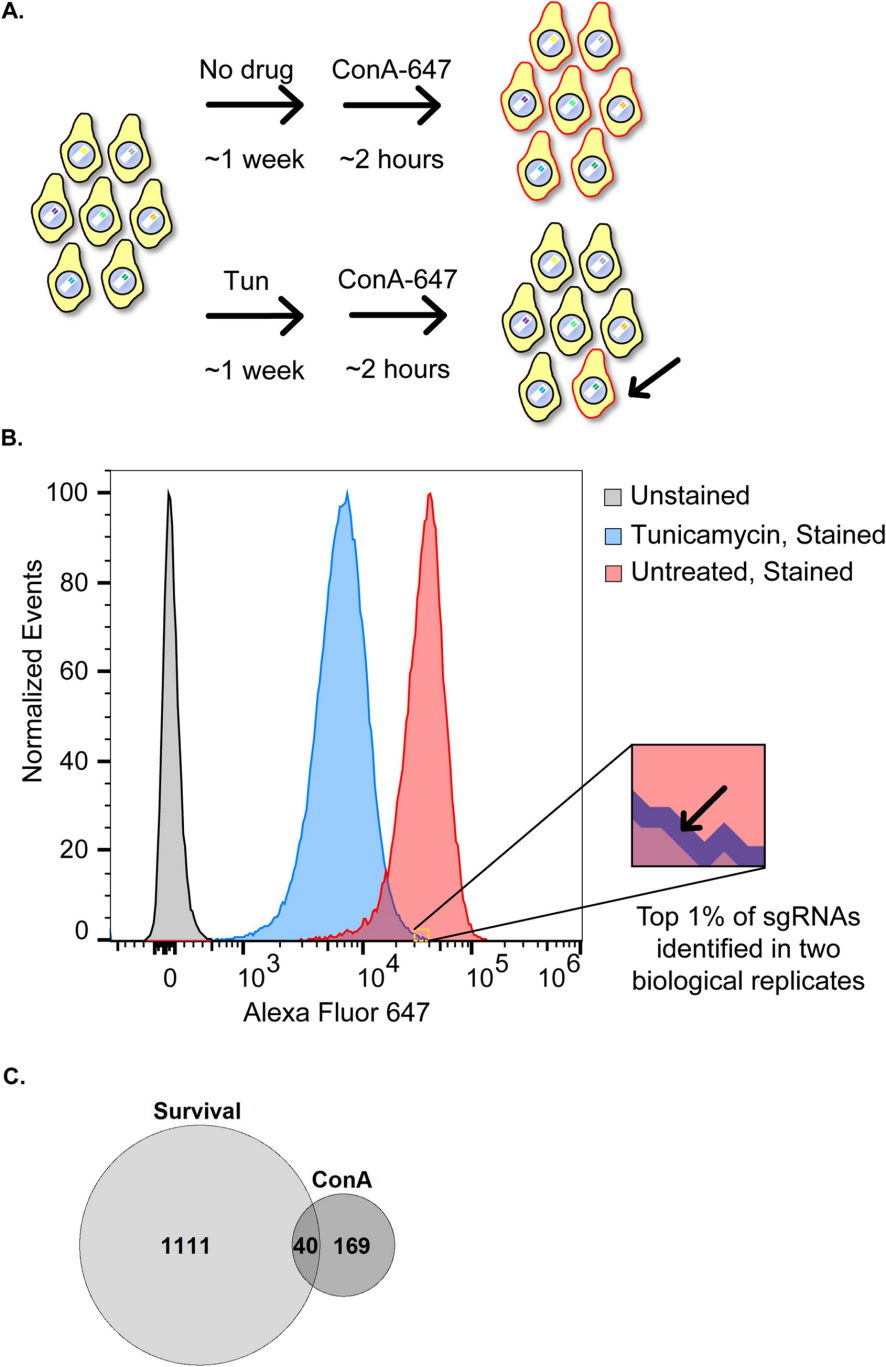
A Concanavalin A screen for gene knockouts capable of restoring cell surface glycoproteins under DPAGT1 inhibition. (A) CRISPR knockout cells were either untreated or treated with Tun, and then stained with fluorescently-labeled ConA to mark cell-surface glycoproteins. (B) Flow cytometry output of the ConA experiment. Tun treatment caused a ~5-fold decrease in ConA staining intensity on average. The "blown-up" section denotes the start of the gate and encompasses Tun-treated cells capable of consistently restoring cell surface glycoproteins to untreated cell levels (the top ~1% of cells). (C) Venn diagram comparison of the survival and ConA screens. There were 40 genes that restored cell surface glycoproteins and provided resistance to DPAGT1 inhibition.

Tun inhibition of DPAGT1 results in reduction of specific glycoproteins from the cell surface such as AchR [30,31] and CASR [32] as well as total cell surface glycosylation [33]. Some candidate modifier genes might rescue the cellular survival phenotype by rescuing cell surface glycoprotein levels. To identify this subset of candidates, we performed a parallel genome-wide screen (Fig 2). We used an identical pool of CRISPR knockout *Drosophila* cells as described above. Again, cells were either untreated or exposed to Tun and fluorescently-labeled with Concanavalin A (ConA), which binds cell surface glycoproteins [34,35]. Cells were then sorted based on fluorescence, with a higher fluorescent signal indicating more cell surface glycoproteins and vice versa. This assay allowed us to determine if a gene knockout can rescue DPAGT1 inhibition-induced loss of cell surface glycoprotein levels. We note that it is possible that some of these knockouts might be capable of restoring cell surface glycoproteins under non-tunicamycin stress conditions.

Under DPAGT1 inhibition, knockout of multiple genes was able to rescue cell surface glycoproteins to the levels observed in untreated cells (S4 Table). We considered genes to be the strongest candidates if they had at least one sgRNA in the highest ~1% of fluorescence of all sgRNAs in two biological replicates (Fig 2 and Tables 4 and S4). This filtering resulted in identification of 209 genes (~1.5% of all genes tested) whose knockout provided strong and consistent rescue of cell surface glycoprotein levels under DPAGT1 inhibition.

**Table 4:** Top 20 candidate genes from the ConA screen.

Fly Gene	Human ortholog	# Guides in top ~1%
*Twin*	*CNOT6L*	10
*CG5276*	*CANT1*	7
*PIG-S*	*PIGS*	6
*PIG-A*	*PIGA*	5
*e(y)3*	*PHF10*	5
*HGTX*	*NKX6-1*	5
*Slif*	*SLC7A1*	5
*Ssh*	*SSH2*	5
*Afti*	*AFTPH*	4
*Bx*	*LMO1*	4
*CG17140*	*VDAC1*	4
*Mvk*	*MVK*	4
*dgt1*	*KANSL2*	4
*Kay*	*FOS*	4
*Mbt*	*PAK4*	4
*Nrg*	*NRCAM*	4
*par-6*	*PARD6G*	4
*PEK*	*EIF2AK3*	4
*Skp2*	*SKP2*	4
*Uzip*	*None*	4

Human orthologs are based on the highest DIOPT score [26].

GO analysis of the 209 genes that restore cell surface glycoprotein levels indicated that "GPI anchor biosynthetic process" (GO:0006506), "glycolipid metabolic process" (GO:0006664), and "inositol metabolic process" (GO:0006020) were the top enriched categories (Tables 5 and S5). Glycosylphosphatidylinositol (GPI) anchor biosynthesis is a form of glycosylation in which a GPI glycolipid is post-translationally attached to proteins in the ER. The first step in GPI anchor biosynthesis involves the attachment of GlcNAc to phosphatidylinositol, and the final GPI-associated proteins are typically transported to membrane-bound lipid rafts [36,37]. Phosphatidylinositol glycan ("*PIG*") genes encode a diverse class of enzymes that build the GPI sugar chain [36,37]. Five different *PIG* genes were among the 209 hits, and all had at least 3 sgRNAs combined from both replicates, placing them in the top ~22% of candidate genes. One of these is *PIG-A* (*PIGA)*, which was also a hit in the survival screen (lfc = 0.81). The other four were *PIG-S* (*PIGS)*, *PIG-B* (*PIGB)*, *PIG-H* (*PIGH)*, and *PIG-O* (*PIGO)*. Notably, these five *PIG* genes are spread throughout the entire GPI anchor biosynthesis process: PIGH and PIGA are both components of the initial GPI-GlcNAc transferase complex that attaches GlcNAc to phosphatidylinositol. PIGB performs an intermediate mannosylation step. PIGO attaches an ethanolamine after PIGB. PIGS is part of the final GPI-transamidase complex which attaches the GPI anchor to a protein. Given their diverse functions, spanning two separate enzyme complexes, it is likely that GPI anchor biosynthesis as a whole can influence the phenotype. Remarkably, the data suggest that impairment of GPI anchor biosynthesis, a process that is typically known to create cell surface glycoproteins, can rescue cell surface glycoprotein levels under DPAGT1 inhibition.

**Table 5:** Gene ontology analysis of candidate genes from the ConA screen.

Gene Ontology	Fold Enrichment	FDR
GPI anchor metabolic process (GO:0006505)	10.82	1.46E-02
liposaccharide metabolic process (GO:1903509)	8.65	1.34E-02
glycolipid metabolic process (GO:0006664)	8.65	1.41E-02
glycolipid biosynthetic process (GO:0009247)	8.52	3.74E-02
membrane lipid metabolic process (GO:0006643)	7.06	2.03E-03
membrane lipid biosynthetic process (GO:0046467)	6.9	7.82E-03
phosphatidylinositol metabolic process (GO:0046488)	6.9	8.53E-03
lipoprotein metabolic process (GO:0042157)	6.31	4.67E-02
glycerophospholipid metabolic process (GO:0006650)	6.07	4.54E-03
glycerolipid metabolic process (GO:0046486)	5.9	1.85E-03

We hypothesized that some genes rescue the cell survival phenotype (Fig 1) by rescuing cell surface glycoprotein levels. To identify these genes, we compared the 209 genes that rescue cell surface glycoprotein levels to the 1151 genes that increase resistance to DPAGT1 inhibition from the survival screen. Strikingly, 40 genes (~19.1% of ConA hits; S6 Table)—including top resistance hits (lfc > 1.5) *PEK*, *Ide*, and *Pfrx*, as well as *PIG-A*—overlapped, which is higher than expected by chance (2.2 fold higher, p<0.0001). Thus, a substantial percentage of gene knockouts that restore cell surface glycoproteins also increase resistance. Nevertheless, as the majority of genes that cause increased resistance did not have this effect (1111 genes, 96.5%), the consistent rescue of cell surface glycoprotein levels is not required to increase resistance to DPAGT1 inhibition.

### Inhibiting the mannosyltransferase Dpm1 rescues *DPAGT1* inhibition and ER stress *in vivo*

**Fig 3:**
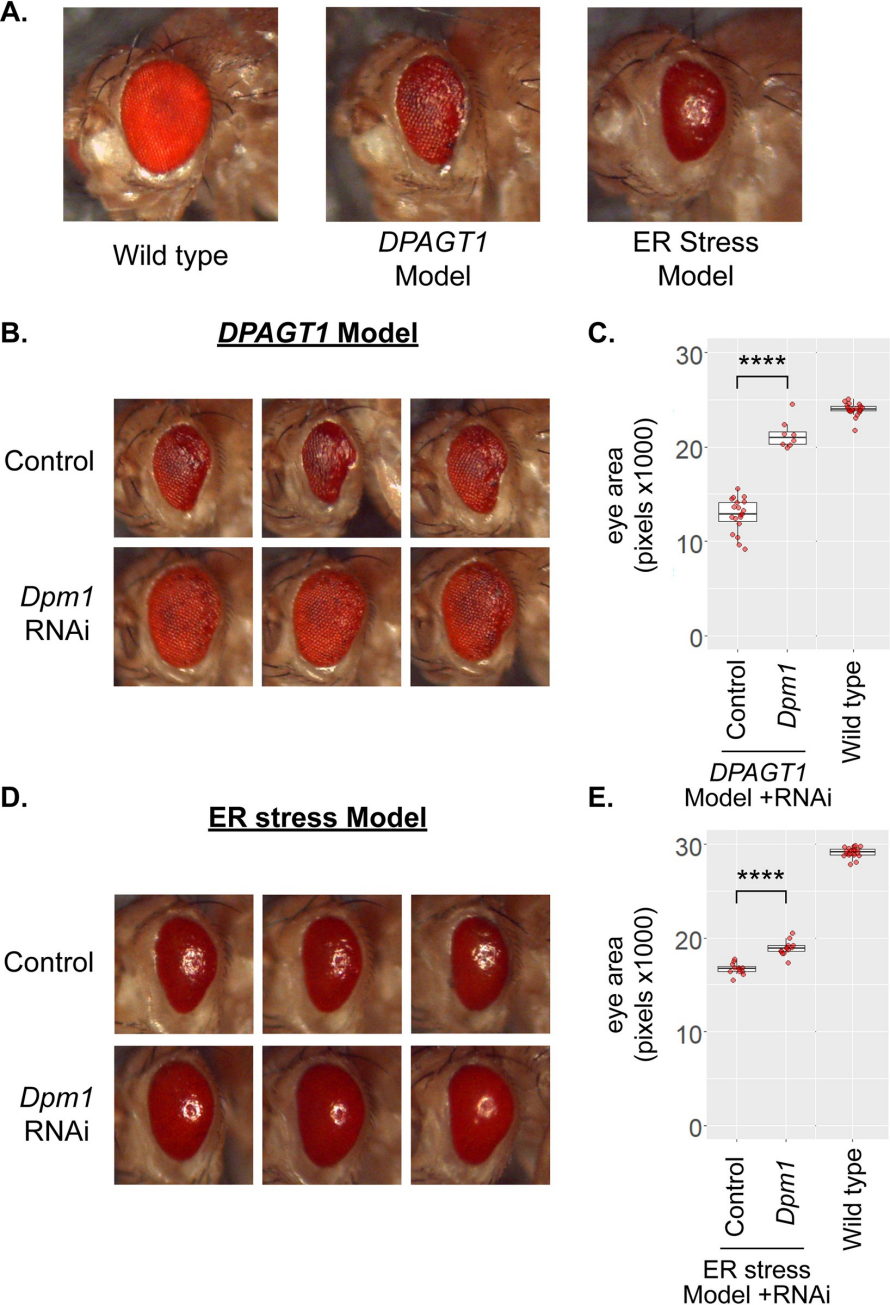
Knockdown of the mannosyltransferase *DPM1* rescues the *DPAGT1* and ER stress models. (A) We generated a *DPAGT1* model that drives RNAi against *DPAGT1* in the fly eye (middle). This causes a small, rough eye phenotype. In addition to the *DPAGT1* model, we also used an ER stress fly model that overexpresses the misfolded protein Rh1^G69D^ in the eye (right). (B) RNAi against the mannosyltransferase *Dpm1* strongly rescues the *DPAGT1* model. The three images are representative from the same experimental cross and RNAi line (BDSC 51396) or control RNAi background (attP2, BDSC 36303). (C) Quantification of *Dpm1* rescue of the *DPAGT1* model. For reference, a representative *DPAGT1* genetically-matched background strain crossed to the control RNAi line is included to indicate the wild type eye size (Wild type).**** p<0.0001 (Student’s t test). (D) *Dpm1* RNAi rescues the ER stress model. Eye images are representative from the same experimental cross and RNAi line (BDSC 50713) or control RNAi background (attP40, BDSC 36304). (E) Quantification of *Dpm1* rescue of the ER stress model. For reference, a representative ER stress genetically-matched background strain crossed to the control RNAi line is included to indicate the wild type eye size (Wild type). **** p<0.0001 (Student’s t test).

To test candidate genes *in vivo*, we created a *DPAGT1* disease model in *Drosophila* that expresses *DPAGT1* RNAi in the eye—causing a small, rough eye phenotype (hereafter referred to as "*DPAGT1* model"; Fig 3A). Initially, we tried using the common GMR-GAL4 eye driver in driving *DPAGT1* RNAi [38]. However, GMR driving *DPAGT1* RNAi surprisingly slightly increased overall eye size (+3.9%, S7 Table) and did not cause obvious eye defects. We believe this difference is due to *eya* expression being both stronger and earlier during development (ModEncode transcriptional data [39,40]). Using the *DPAGT1* model, we can determine the impact of knocking down candidate genes with RNAi by measuring changes in the eye phenotype both quantitatively by size and qualitatively by observing visible phenotypes such as necrosis. We hypothesized that RNAi knockdown of candidate genes from the previous cell screens, that improved phenotypes under DPAGT1 inhibition, would improve the eye phenotype in the *DPAGT1* model. We primarily focused on top significantly-enriched GO categories (FDR<0.05) from the survival and ConA screen (Tables 3 and 5). For validation, we tested multiple RNAi lines against the glycolytic, nucleotide sugar synthesis, GPI anchor pathways, the adjacent GDP-mannose-related pathway, as well as two top resistance hits relating to the ER (S7 Table). We used multiple RNAi lines against each gene when available and used RNAi against multiple genes in the same pathway throughout (see S7 Table for complete RNAi line information).

Many of these candidate genes had *in vivo* effects that were consistent with the outcome from the cell screens, but others had no effect or even the opposite effect (S7 Table). However, some of the strongest candidate genes from the cell screen had a consistent effect in the *DPAGT1* model (S7 Table). For example, two of the top resistance genes from the survival screen increased eye size: *PEK* (*PERK*) (+14.7% and +27.4%; lfc = 4.76, ConA screen hit) and *Hrd3* (*SEL1L*) (+33.8% and +12%, lfc = 3.7).

We also tested *Pfk* (lfc = 1.47) and *Pfrx* (lfc = 1.74), as they have the highest lfc values in the glycolytic pathway from the survival screen. *Pfrx* was also a hit in the ConA screen (S4 Table). Matching the strong effect that we observed in the survival screen, RNAi against either of these two enzymes improved the *DPAGT1* model (*Pfk*: +32.2%, *Pfrx*: +11.3%, averaged across two biological replicates). On the other hand, in the nucleotide sugar synthesis pathway, RNAi against transaminase-encoding *Gfat2*, despite causing resistance (lfc = 4.68), did not significantly affect the *DPAGT1* model (S7 Table). Moreover, RNAi against the deaminase-encoding *Oscillin*, which caused sensitivity (lfc = -1.87), improved the *DPAGT1* model (+28.8%). These differences from the cell screen may be caused by the differing biology of simple replicating cells compared to an *in vivo* eye model which has to pass through multiple stages of development and differentiation.

We tested RNAi against genes encoding four enzymes in GDP-mannose synthesis, Pmm2, Mpi, CG3792, and Dpm1. One RNAi line against *Pmm2* rescued the *DPAGT1* model eye size (+22.2%, S7 Table). *Mpi* RNAi worsened eye size (-9.7%, S7 Table), though we note that Mpi catalyzes a reversible reaction in GDP-mannose synthesis. RNAi against *CG3792* (*MPDU1*), important for bioavailability of downstream dolichol phosphate mannose (Dol-P-Man) [41,42], did not affect *DPAGT1* model eye size (S7 Table and S3 and S4 Figs). However, two RNAi lines against the mannosyltransferase *Dpm1* (*DPM1;* lfc = 0.68) showed the strongest rescue of the *DPAGT1* model phenotypes (+62.1%; +82.5%) (Fig 3B and 3C and S7 Table and S1 and S2 Figs). *Dpm1* encodes dolichol-phosphate mannosyltransferase 1, which, in complex with the membrane-anchored proteins DPM2 and DPM3, synthesizes Dol-P-Man, an essential substrate in N-glycosylation, O- and C-mannosylation, and GPI anchor biosynthesis [43–46] (Fig 1C). Of the two anchor proteins, no RNAi is currently available for *Dpm3* in flies. RNAi against *Dpm2* had inconsistent but slightly negative effects in the *DPAGT1* model (-7.5%, S7 Table and S3 and S4 Figs). The results on *MPDU1* and *Dpm2* match the cell screen data where neither of these genes were hits, and it suggests that specific loss of the catalytic domain of the DPM1 complex (*Dpm1*) is most critical for improving *DPAGT1* deficiency. In sum, while loss of *DPM1* is normally detrimental to cells and patients [12,44,47], knockdown of *Dpm1* improved the *DPAGT1* model.

To determine if the *Dpm1* rescue was specific to *DPAGT1* or ER stress in general, we also tested each RNAi line on the well-established *Drosophila* Rh1^G69D^ ER stress model [48–51] (hereafter referred to as "ER stress model") (Fig 3A and S7 Table). The ER stress model overexpresses a misfolded protein in the eye in order to induce ER stress, apoptosis, and a small, rough eye phenotype. Similar to the *DPAGT1* model, *Dpm1* RNAi resulted in the strongest rescue of overall eye size in the ER stress model (+18%, Fig 3D and 3E and S7 Table, and S1 and S2 Figs), indicating that *Dpm1* inhibition is capable of rescuing the effects of *DPAGT1* inhibition and ER stress in general. We also note that *Dpm1* RNAi did not significantly impact eye size in either control driver line alone, except for one line in the ER stress control where it slightly reduced eye size (-3%, S7 Table). Despite the fact that *Dpm1* is an essential enzyme, inhibiting *Dpm1* provides significant benefits to *DPAGT1* inhibition and ER stress, and thus, it may be a key factor in rescuing the effects of *DPAGT1* deficiency.

### Impairment of O-mannosylation, N-glycosylation, and GPI anchor biosynthesis improves the loss of *DPAGT1* function and ER stress outcomes

One hypothesis for how *Dpm1* inhibition might rescue *DPAGT1* inhibition and ER stress is through the reduction of its product, Dol-P-Man, and the subsequent impairment of Dol-P-Man-dependent glycosylation pathways [46,52]. This hypothesis fits with other screen data. For example, loss of one downstream pathway of Dpm1, GPI anchor biosynthesis, improves cell survival (genes: *PIG-A*, lfc = 0.81; *PIG-T*, lfc = 0.57; *PIG-K*, lfc = 0.50; *PGAP1*, lfc = 0.61) and rescues cell surface glycoproteins (genes: *PIG-A*; *PIG-H*; *PIG-O*; *PIG-B*; *PIG-S*) (Figs 1 and 2 and S1 and S4 Tables). To test this hypothesis, we crossed strains expressing RNAi against the mannosyltransferase enzymes in O- and C-mannosylation, N-glycosylation, and GPI anchor biosynthesis to the *DPAGT1* model (S3 and S4 Figs).

**Fig 4:**
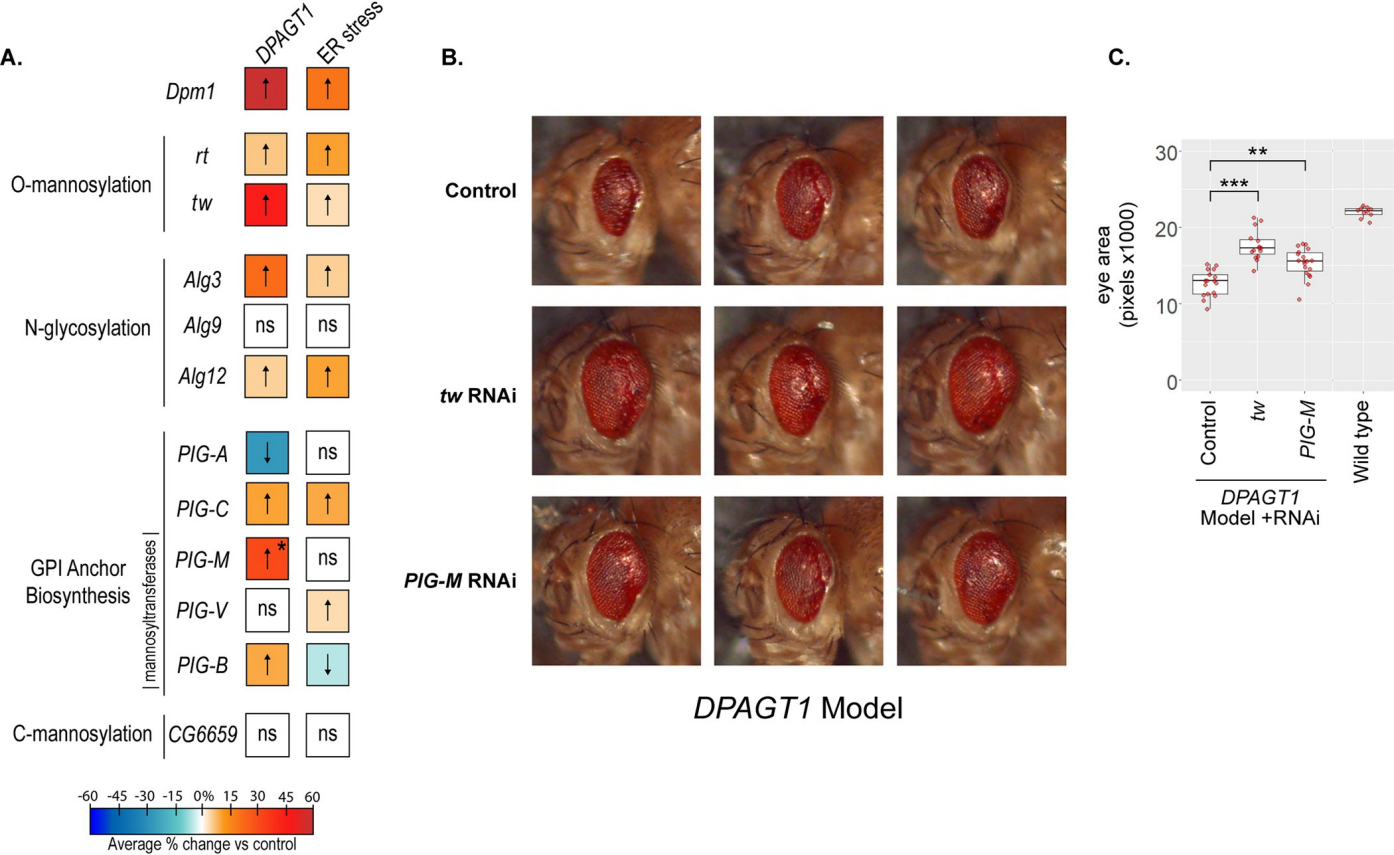
Knockdown of glycosylation pathways rescues the *DPAGT1* and ER stress models. (A) A summary of RNAi data performed on downstream DPM1 glycosylation pathways. The majority of mannosyltransferases tested in O-mannosylation, N-glycosylation, and GPI anchor biosynthesis pathways increased eye size in the *DPAGT1* model. Colors denote % change of eye size vs their control; warmer colors (or up-arrows) have a stronger effect, and vice versa. Colors are derived from the averages of at least 2 biological replicates with at least one significant replicate and no opposite results (see S7 Table for all RNAi data). * = One *PIG-M* RNAi line was very strong (BDSC 51890), while a second line was neutral/negative in response (see S7 Table). (B) Representative images of the *DPAGT1* model crossed with RNAi against the two strongest hits, *tw* and *PIG-M*. Shown are three representative images from the same RNAi line and experimental cross (*tw*: BDSC 55735, *PIG-M*: BDSC 51890) or control RNAi background (attP40: BDSC 36304). (C) Quantification of *tw* and *PIG-M* rescue shown in B. For reference, the total quantification of that experiment, as well as a representative wild type *DPAGT1* genetically-matched background strain crossed to the control RNAi line, is included. ** p<0.01, *** p<0.001 (Student’s t test).

O-mannosylation begins with the addition of mannose to Ser/Thr residues, followed by a lengthening of the chain with additional sugar molecules [53,54]. One important O-mannosylated protein is alpha-dystroglycan, and impairment of O-mannosylation is associated with muscular dystrophy in humans [54]. The first step in O-mannosylation, which attaches the initial mannose from Dol-P-Man, requires an enzyme complex consisting of two O-mannosyltransferases, POMT1 and POMT2. RNAi against *rotated abdomen* (*rt*, *POMT1*) or *twisted* (*tw*, *POMT2*) rescued eye size in both the *DPAGT1* (*rt*: +6.2%; *tw*: +45.9%) and ER stress (*rt*: +10.2%; *tw*: +3.8%) models (Fig 4A–4C and S7 Table and S3 and S4 Figs). These data suggest that loss of O-mannosylation can rescue the loss of *DPAGT1* function and ER stress.

In N-glycosylation, mannosylation steps utilizing Dol-P-Man occur in the ER lumen via the mannosyltransferases ALG3, ALG9, and ALG12 [55]. RNAi against *Alg3* (*ALG3*) and *Alg12* (*ALG12*) improved eye size in both the *DPAGT1* (*Alg3*: +21.4%; *Alg12*: +4.5%) and ER stress (*Alg3*: +4.8%; *Alg12*: +9.9%) models. However, RNAi against *Alg9* (*ALG9)* did not affect either model (Fig 4A and S7 Table and S3 and S4 Figs). Overall, loss of the N-glycosylation mannosyltransferase steps can rescue the loss of *DPAGT1* function and ER stress.

In the GPI anchor biosynthesis pathway, most RNAi lines tested improved the *DPAGT1* model. These include a subunit of the GPI-GlcNAc transferase complex, *PIG-C* (*PIGC*, +22.6%), and the mannosyltransferases *PIG-B* (*PIGB*, +11.3%) and *PIG-M* (*PIGM*, +31.9%). A second *PIG-M* RNAi line had a small negative effect (-5.7%). RNAi lines against the mannosyltransferase *PIG-V* (*PIGV*) did not have a significant effect. Surprisingly, RNAi against *PIG-A* was detrimental to the *DPAGT1* model (-5.7% and -27.7%, respectively). Unlike the previous two pathways tested, the ER stress model was not as consistent with the *DPAGT1* model, though it also had an overall beneficial effect (Fig 4A–4C and S7 Table and S3 and S4 Figs). This may reflect a difference in how GPI anchor biosynthesis affects each model.

C-mannosylation is a less common type of glycosylation that involves the addition of mannose to tryptophan found in thrombospondin type 1 repeats [56]. The four enzymes in humans responsible for C-mannosylation are *DPY19L1-4* (*DPY19L1* and *DPY19L3* being the most functionally important [56]). In *Drosophila*, *CG6659* is the single ortholog of all four human paralogs (DIOPT scores for *DPY19L1-4*: 10/16, 9/16, 4/16 and 4/16). RNAi against *CG6659* did not affect eye size in either model; however, here we note an increased chance of a false negative as only one RNAi line and one gene from this pathway were examined (Fig 4A and S7 Table and S3 and S4 Figs). Thus, it is not immediately clear that impairing C-mannosylation imparts any benefit under the loss of *DPAGT1* function or ER stress.

Taken together, disruption of any of the three pathways downstream of Dpm1 and its product Dol-P-Man—O-mannosylation, N-glycosylation, and GPI anchor biosynthesis—can rescue the effects of *DPAGT1* inhibition and ER stress. Notably, RNAi against any single gene in these three pathways never reached the same magnitude of rescue provided by *Dpm1* RNAi (+62.1%)—with the closest being *tw* (+45.9%) and *PIG-M* (+31.9%) RNAi (Fig 4B and 4C). This suggests that the strong rescue of the *DPAGT1* model with *Dpm1* RNAi may be from the synergy of the combined impairment of the three downstream glycosylation pathways. In addition, for every gene tested, RNAi downregulation in the *DPAGT1* model had stronger, more consistent rescue of negative phenotypes than the ER stress model, which suggests an underlying difference between the two models.

### Fructose metabolism is differentially altered depending on the source of ER stress

Functions related to glycolysis and metabolism were enriched among our candidate genes (S5 Fig and Tables 3 and S3). In the survival screen (Fig 1), knockout of glycolytic genes generally caused resistance to *DPAGT1* inhibition, including the aforementioned enzymes *Pfk* (lfc = 1.47) and *Pfrx* (lfc = 1.74), as well as the dehydrogenase *Gapdh1* (*GAPDH*; lfc = 1.14), phosphoglycerate mutase *Pglym87* (*PGAM2*; lfc = 1.21), and pyruvate kinase *CG12229* (*PKM;* lfc = 0.73), among others (S1 Table and S5 Fig). Loss of *Pfrx* also improved cell surface glycoproteins in the ConA screen (Fig 2). Thus, impairing glycolysis appears to improve survival under the loss of *DPAGT1* function.

**Fig 5:**
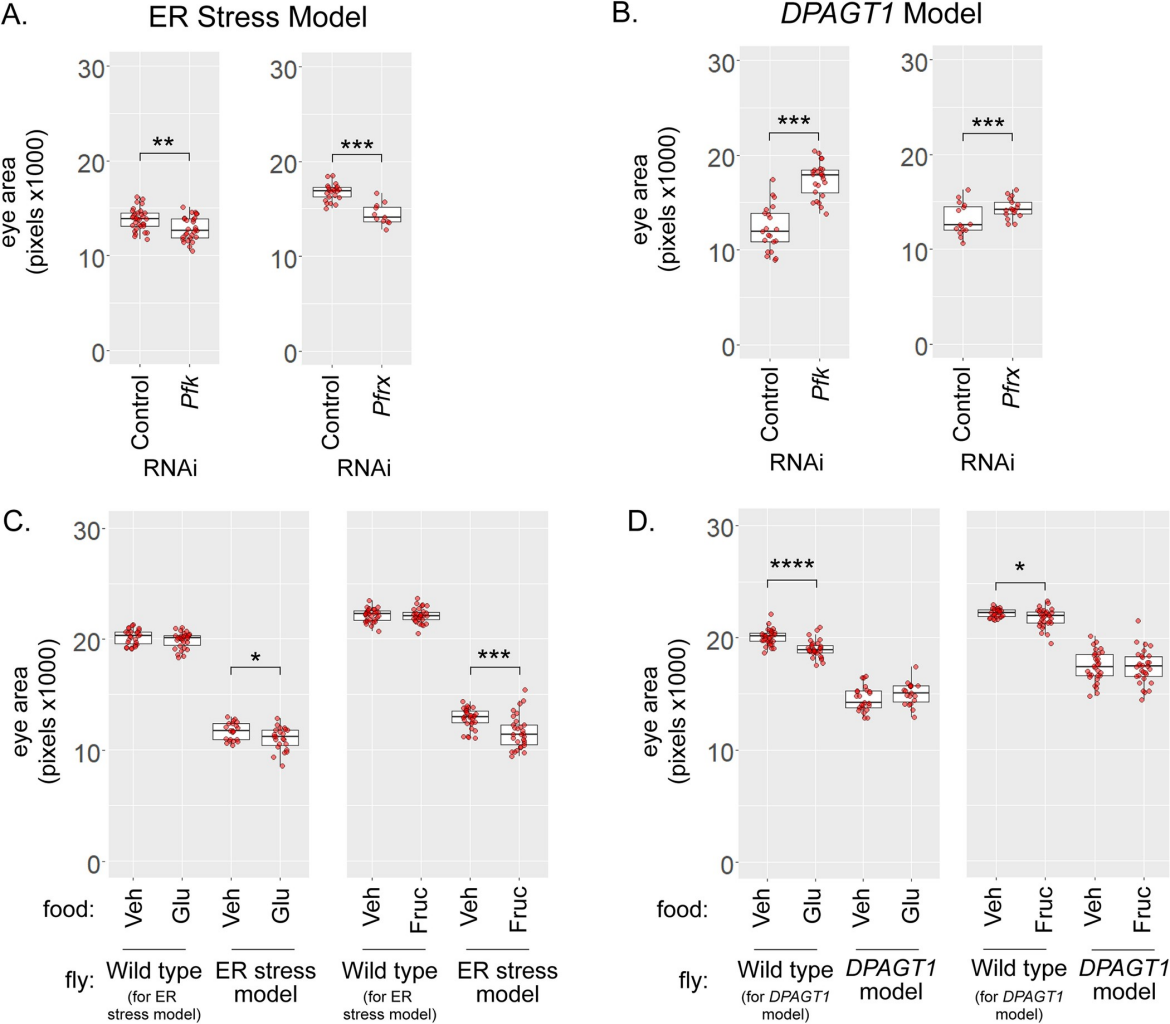
Glucose and fructose metabolism differences between the *DPAGT1* and ER stress models. (A-B) In the ER stress model, knockdown of *PFKM*/*Pfk* (BDSC 34336) and *PFKFB1*/*Pfrx* (BDSC 57222) causes a worsening of eye size compared to control RNAi (attP2, BDSC 36303; and attP40, BDSC 36304). In contrast, in the *DPAGT1* model, knockdown of these genes causes an overall improvement of eye size. (C) 100mM dietary glucose or fructose significantly reduced eye size in the ER stress model. Wild type refers to the genetically-matched background strain. (D) Compared to its background control, feeding of 100mM glucose or fructose was net-positive in the *DPAGT1* model. Wild type refers to the genetically-matched background strain. For A-D, 1 replicate is shown of 2 biological replicates (S8 Table), * p< 0.05, ** p<0.01, *** p<0.001 (Student’s t test).

Surprisingly, knockdown of *Pfk* and *Pfrx* worsened the ER stress model (Fig 5A and S7 Table). In agreement with the screen data, knockdown of either of these two enzymes improved the *DPAGT1* model (Fig 5B and S7 Table). Pfk and Pfrx both act on the early glycolytic metabolite fructose-6-phosphate to regulate glycolysis (Fig 1C). While Pfrx can have phosphatase activity, one potential hypothesis is that loss of Pfk and Pfrx function might generally increase the pool of fructose-6-phosphate in the cell. Given the shared substrate and differential effects in the two models, one possible reason could be key differences in how fructose-6-phosphate levels and altered metabolism affects the loss of *DPAGT1* function and ER stress.

Typically, glucose is metabolized into fructose-6-phosphate using two enzymes, hexokinase and phosphoglucose isomerase [57]. In addition, while in most cases fructose is converted into fructose-1-phosphate by fructokinase, it can also be synthesized directly into fructose-6-phosphate using hexokinase [58–61]. Given that glucose or fructose can be metabolized into fructose-6-phosphate, we tested whether these sugars could differentially affect the *DPAGT1* and ER stress models through dietary supplementation. In agreement with the RNAi data, feeding the ER stress model flies dietary glucose or fructose led to a significant decrease in overall eye size (-8.9% and -9.7%, averaged from two biological replicates) (Fig 5C and S8 Table). In contrast, its genetically-matched control was not affected by glucose or fructose supplementation. In the *DPAGT1* model, dietary glucose or fructose had no significant reproducible effects on eye size. In contrast, its genetically-matched control had negative effects on eye size from glucose and fructose (-3.0% and -2.4%) (Fig 5D and S8 Table). While the ER stress model showed a net negative effect, compared to its genetically matched control, from sugar supplementation, the *DPAGT1* model had an overall net positive effect. Together, the RNAi and sugar feeding data reveal a difference in glycolytic metabolism between the two models.

## Discussion

Here, we report hundreds of new candidate genes that modify the effects of *DPAGT1* inhibition identified using a cell-based CRISPR screen. Testing these candidate genes *in vivo* validated many of the strongest hits and demonstrated that knockdown of GPI anchor biosynthesis and downstream *DPM1*/*Dpm1* glycosylation pathways can rescue the effects of *DPAGT1* deficiency and ER stress. Even though single gene mutations in these pathways are associated with specific CDGs, knockdown of many of these genes paradoxically improved negative phenotypes associated with *DPAGT1* inhibition and ER stress both *in vitro* and *in vivo*.

One hypothesis for this outcome is that impairing glycosylation pathways might slow down the overall synthesis of glycoproteins, thereby allowing cells to better withstand *DPAGT1* inhibition. Similar mechanisms are utilized in the response to ER stress, where the UPR will reduce protein synthesis in response to ER stress, giving the cell time to recover [5,62]. Another mechanism by which impairing glycosylation may rescue the loss of *DPAGT1* function is by freeing up cellular machinery that helps fold or degrade misfolded proteins. One such pathway is the calnexin/calreticulin (CNX/CRT) cycle, a process that ensures the proper folding of N-glycosylated proteins in the ER [63]. Recently, the CNX/CRT cycle was found to help mature GPI-associated proteins as well [64]. In one scenario, it is possible that impairing the GPI anchor biosynthesis pathway helps free up machinery in the CNX/CRT cycle, allowing it to fix or clear more misfolded N-glycosylated proteins under loss of *DPAGT1* function. ER-associated degradation (ERAD) involves the recognition, translocation, and ubiquitination/degradation of misfolded proteins in the ER through the use of different enzyme complexes [65]. One component of the ERAD Hrd1 complex is the adaptor subunit, Hrd3 (human: SEL1L) [66]. *Hrd3* was a strong hit in the cell survival screen (Table 2), and it also strongly rescued the *DPAGT1* model (S7 Table). Deficiencies of HRD1 or SEL1L can lead to accumulation of proteins in the ER [67]. As such, one potential hypothesis is that loss of *Hrd3* impairs the Hrd1 complex, causing more retention of misfolded proteins in the ER and allowing them longer to properly fold (potentially by the aforementioned CNX/CRT cycle). Finally, as *Hrd3* RNAi only improved the *DPAGT1* model, but had either no effect or worsened the ER stress model, this suggests another difference between the mechanism of stress between each model and how that stress may be ameliorated. Overall, this might provide a novel approach to DPAGT1-CDG or other glycosylation disorders, where a therapy might be designed to slow down global glycosylation and/or globally activating proteostasis machinery, to allow impacted cells more time to repair or clear misfolded proteins.

Because independent perturbations of three downstream Dpm1-dependent glycosylation pathways alone were capable of partially rescuing the *DPAGT1* model, each one could provide a good therapeutic target for DPAGT1-CDG. Although targeting *DPM1* itself might be a useful approach, targeting a downstream gene may be better in practice to reduce the effects of elimination of all glycosylation pathways. For example, knockdown of *POMT2/tw* almost reached the same level of rescue as *Dpm1* alone (+45.9% vs. +62.1%), while targeting *POMT2* would perturb only O-mannosylation. O-mannosylation affects cadherins, plexins, and alpha-dystroglycan, among other proteins [68], and is associated with multiple CDGs, including POMT2-CDG [69]. Thus, like many other candidate genes identified in our study, care would need to be taken in inhibiting this gene in any potential therapies. Nevertheless, given the multiple target genes throughout these pathways, this approach could allow for better "titration" of genetic or pharmacologic downregulation, which could hopefully provide an effective therapeutic approach. One other hypothesis is that inhibiting *Dpm1* might increase the pool of dolichol-phosphate available for DPAGT1 to utilize (including under inhibition). While this would not explain why inhibiting glycosylation pathways downstream of *Dpm1* also improves *DPAGT1* inhibition, it could be partially responsible for why loss of *Dpm1* alone causes stronger rescue than loss of any of its downstream pathways. Alternatively, another hypothesis is that *DPAGT1* inhibition may increase flux of dolichol-phosphate through Dpm1 to cause excessive glycosylation, which is then turned off under Dpm1 inhibition in order to cause its rescue effect. However, given that tunicamycin strongly reduced binding of ConA, which binds to mannose/glucose moieties in glycoproteins [70,71], it is unlikely that there is excessive glycosylation under DPAGT1 inhibition.

RNAi against *Mpi* (*MPI*), another gene in the GDP-mannose pathway, worsened the *DPAGT1* and ER stress models (S7 Table). However, MPI interconverts mannose-6-phosphate and fructose-6-phosphate [72], which means its inhibition could increase or decrease GDP-mannose synthesis. Given that we also see differential effects of fructose in our two models, metabolomic analysis of these pathways may be required to understand the exact mechanism of *Mpi* loss under *DPAGT1* impairment. Of note, *Mpi* was a resistance hit in the cell survival screen, which suggests there could be differences in metabolite utilization between the *in vitro* and *in vivo* models. Surprisingly, loss of neither *Dpm2*, encoding a protein important for anchoring DPM1, nor *CG3792*, encoding a protein important for utilizing Dol-P-Man, could improve the *DPAGT1* model. Neither gene was a hit in the cell screens, and this suggests it may be more important to directly impair the synthesis of Dol-P-Man—as loss of two other enzymes in the GDP-mannose pathway, *Pmm2* and *Dpm1*, both improve the *DPAGT1* model (S7 Table). However, one hypothesis is that there may be another pathway in the fly capable of increasing availability of Dol-P-Man, or that *Dpm1* may have some other function, beyond its enzymatic activity, that is important in regulating its downstream glycosylation pathways.

RNAi against many of the GPI anchor biosynthesis genes improved one or both of the *in vivo* models. However, despite being a hit in both cell screens, *in vivo* knockdown of *PIGA/PIG-A* caused a reduction in eye size in the *DPAGT1* model and no change in the ER stress model. We hypothesize that *PIGA/PIG-A* may be too critical for development *in vivo*, such that developmental effects mask potential protection in either model. In support of this hypothesis, *PIGA/PIG-A* knockdown alone results in more severe eye phenotypes compared to other *PIG* genes (S3 and S4 Figs). For example, *PIG-B*, another PIG gene hit from the ConA screen, was capable of improving the *DPAGT1* model, but had a milder phenotype when knocked down alone. There may be a limit on how strongly these downstream pathways can be inhibited before effects change from beneficial to deleterious.

The strongest candidate gene from the cell screen with improved survival under *DPAGT1* inhibition was *PEK* (*PERK*), which also rescued cell surface glycoproteins in the ConA screen. *PEK* knockdown also improved the *in vivo DPAGT1* model. *PEK* encodes a protein kinase essential to the cellular ER stress response. Other genes encoding ER stress sensor proteins, *Xbp1* and *ATF6* [7], did not reach our LFC threshold (lfcs = -0.48 and 0.33, respectively). PEK activates the transcription factor Crc (ATF4), which can regulate both pro-survival or apoptotic-related genes [7]. One hypothesis for why knockout of *PEK* leads to increased survival is that ER-stress-induced apoptotic-related gene expression might be lower in these cells, which may allow them time to grow and repair despite being stressed. While *Drosophila* lacks the ATF4 downstream pro-apoptotic target CHOP, *Drosophila* Crc can activate apoptosis by downregulation of the anti-apoptotic E3 ubiquitin ligase XIAP/Diap1 [73]. Similar to *PERK*, knockout of *crc* does provide resistance (lfc = 0.75). Interestingly, Crc is also a co-activator of the hormone receptor FXR/EcR [74], and signaling of the hormone ecdysone through this receptor is critical for proper morphogenesis in the fly [75,76]. Knockout of *FXR/EcR* also provides resistance (lfc = 2.9). Thus, part of the resistance from *PEK* and *crc* knockout might result from its connection to ecdysone signaling.

The majority of genes for which knockout increased resistance to Tun did not overlap with hits from the cell surface glycoprotein ConA screen (S6 Table). This suggests that rescue of surface glycoproteins is not required for cell survival. However, many ConA gene hits did overlap, such as *PIG-A*, *Pfrx*, and *PEK*, which may hint at the mechanisms through which these gene pathways lead to survival. It is possible that loss of a specific protein leads to both Tun resistance and cell surface glycoprotein rescue in these overlapping genes. For example, perturbing GPI anchor biosynthesis genes can improve cell survival, cell surface glycoproteins, and *in vivo* models of *DPAGT1* inhibition, which could indicate that loss of a specific GPI-anchored protein improves these phenotypes. As a different example, loss of *PEK* also improves these metrics under *DPAGT1* inhibition, which suggests the importance of ER stress and/or apoptosis to these phenotypes given the role of *PEK* noted above [7]. As all gene knockouts occur independently of each other in the CRISPR screens, it is also possible that both of these mechanisms are true in any individual knockout cell.

Not every gene from the cell screens validated *in vivo*. We believe this is primarily driven by differences in the biology of single cells vs. whole, developing organisms, but it is also possible some opposite results are driven by false discovery. In CRISPR knockout screens, false discovery can be due to off-targeting, poor guide RNA efficiency, or incomplete knockout [77–79]. We employed 4–8 guide RNAs per gene, while utilizing the median LFC value for downstream analysis—both of which should improve the overall false hit rate [78]. However, it is still possible that false positives occur even when multiple guide RNAs are used [77]. For example, the gene *Diap1* was a strong hit in our screen, yet loss of *Diap1* alone is known to kill S2 cells [80], so this is likely a false positive. We had 8 final guides for *Diap1*, and 6 of them were in enriched at LFC = 0.5 in the Tun-treated population. As each guide is unique, it is unlikely this is being driven by off-targets (though it may partially explain this result). One possibility is that, in rare circumstances, some cells have only single allelic knockout of *Diap1*, and that this heterozygosity may improve Tun survival.

There are several other candidate genes that might be informative for understanding *DPAGT1* deficiency. For example, another enriched GO pathway from the ConA screen was "inositol metabolic process" (GO:0006020) (Table 3). Inositol has several functions, including growth, immunity, and GTPase signaling [81]. One of these functions may play an important role in *DPAGT1* inhibition, unrelated to glycosylation. Other GO enriched categories of note include nucleotide synthesis (Table 3). Activation of the UPR transcription factor ATF4 can promote *de novo* purine synthesis through the tetrahydrofolate cycle [82,83]. Perhaps modulation of purine synthesis might provide some benefit under *DPAGT1* inhibition.

We also found that sugar supplementation had a different effect on the *DPAGT1* and ER stress models, and this might be explained by the disease mechanism of each model. The *DPAGT1* model ostensibly works by reducing the amount of functional DPAGT1 enzyme (similar to Tun treatment), and DPAGT1 enzymatic activity utilizes UDP-GlcNAc, which is a product of fructose-6-phosphate metabolism through the hexosamine pathway (Fig 1C). Inhibition of DPAGT1 enzymatic activity may result in a complex pattern of feedback that affects both fructose-6-phosphate levels and metabolism. While impairment of this enzyme in cells is associated with ER stress [12,18], it is possible the rough eye phenotype is induced by impaired trafficking or the loss of a specific glycosylated protein. In contrast, the ER stress model overexpresses a single misfolded protein, causing accumulation in the ER lumen and subsequent ER stress [49]. The ER stress model does not directly impair the fructose metabolism pathway, and its misfolded protein landscape is likely dominated by a single misfolded protein. Given these differences, we hypothesize that altering fructose-6-phosphate metabolism may have a differential effect on DPAGT1 because of its metabolic connection to the hexosamine pathway. Metabolomic analysis should be performed to further test this hypothesis—especially considering that the related metabolite mannose-6-phosphate is associated with ER stress [84]; while metabolomic analysis of the fly eye disc is technically challenging due to a low number of relevant cells, this could be further tested in *DPAGT1* mutant cell lines [18].

In this study, we used cell culture and *in vivo* approaches to identify hundreds of new modifier genes affecting *DPAGT1* inhibition. Strikingly, we found that Dpm1 and its downstream glycosylation pathways are major enriched sites of these modifier genes. Our screen highlights that genes individually associated with one CDG may also be a source of modifiers of another CDG. If found to be more generally true, this paradoxical relationship between CDG genes could be exploited for therapeutic benefit. Targeting individual parts of these pathways under careful titration with drug or gene therapy may be the answer to better treatments for DPAGT1-CDG. In addition, we found differences in fructose metabolism that may explain differences between models of disease and ER stress and could be key to developing future dietary treatments for this disorder. Taken together, we believe these findings serve as a staging ground for further elucidation of modifier genes of *DPAGT1* inhibition and how they affect its underlying metabolism.

## Materials and methods

### Cell culture

*Drosophila melanogaster* S2R+ cells are from the Drosophila RNAi Screening Center (Harvard Medical School). A subline expressing SpCas9 and containing an attP integration site, S2R+/NPT005/MT-Cas9 (PT5/Cas9), was described previously [25,85] and is available at the Drosophila Genomics Resource Center (Cell line # 268). Cells were maintained in Schneider’s media (Thermo 21720) supplemented with 1X Penn/Strep (Thermo 15070063) and 10% FBS (Thermo 16140071).

### Cell toxicity assay for tunicamycin sensitivity

Tunicamycin (Cayman Chemical # 11445) dissolved in DMSO was serially diluted 2-fold into growth media in 96-well plates. Then, cells were added such that each well contained 10,000 cells in 100 μL. Plates were incubated at 25°C for 4 days and then relative cell titers were determined using Cell Titer Glo (Promega) and a luminescence plate reader (SpectraMax) according to the manufacturer’s instructions.

### Genome-wide CRISPR–Cas9 screening for resistance to tunicamycin

The *Drosophila* CRISPR genome-wide knockout library was previously described and is available from Addgene (134582–4) [25,85]. In brief, PT5/Cas9 cells were transfected with an equal-parts mixture of pLib6.4 containing an sgRNA library as well as pBS130 (26290, Addgene) using Effetene (301427, Qiagen) following the manufacturer’s instructions. The library was delivered in three parts, sublibrary group 1, 7,956 gRNAs for 995 genes; sublibrary group 2, 17,827 gRNAs for 2979 genes; and sublibrary group 3, 59,406 gRNAs for 9954 genes. After 4 days, the transfected cell library was selected with 5 μg/mL puromycin (540411, Calbiochem) for 12 additional days, subculturing every 4 days. After the stable sgRNA*-*expressing cell library was established, 1000 cells per sgRNA were subcultured in each passage to maintain the expected diversity of the sgRNA library. The cells were exposed to 590 nM tunicamycin (500 ng/ml, Cayman Chemical # 11445) for 30 days, passaging the same number of cells (~8×10^7^) to four new 15 cm dishes every 4 days. Following the final passage, aliquots of the cells were collected, and their genomic DNA was extracted using a Zymo Quick-gDNA Miniprep kit (D3025, Zymo Research). DNA fragments containing the sgRNA sequences were amplified by PCR using at least 1000 genomes per sgRNA as template for each sample. The in-line barcoding strategy and sequences were as previously described [85]. Next generation sequencing (Illumina NextSeq) was performed at the Biopolymers Facility at Harvard Medical School. Following barcode demultiplexing, a readcount table was prepared from the raw sequencing files using MAGeCK version 0.5.9.4 [86], *count* subprogram, using standard parameters with 22 bp 5’ trimming and median-normalizing each sublibrary to 10,000 reads. The median log2 fold-change reported throughout was calculated from this data directly by measuring log2 of treated readcount divided by untreated readcount and then determining the median value among all sgRNAs targeting the same gene. For the volcano plot, the log2 fold-change and robust rank aggregation (RRA) p-value measures were determined using MAGeCK version 0.5.9.4 software, *test* subprogram, using standard parameters.

### FACS-based selection for altered concanavalin A staining intensity

After generating puromycin-resistant pools of cells expressing a genome-wide library CRISPR sgRNA library (88,627 sgRNAs targeting 13,685 *Drosophila* genes), the cells were again subjected to treatment with tunicamycin (590 nM) in normal growth medium for 1 week. Next, 1×10^8^ cells were placed into suspension in 10 mL and labeled live with 10 μg/mL Alexa Fluor 647 Conjugated Concanavalin A (Thermo # C21421) for at least 2 hours by diluting the stock 1:500 into full growth medium at room temperature with occasional inversion. 1 mL aliquots of labeled cells were transferred individually to 5 mL 40 μm filter-cap vials to mechanically declump the cells. Cells were then subjected to fluorescence-activated cell sorting (FACS) using a Sony MA900 FACS machine guided by gates established by tunicamycin-untreated cells stained in parallel and unstained cells. Approximately 1% of cells that exhibited the greatest intensity of staining in the AlexaFluor 647-A filter (corresponding to the average intensity of tunicamycin-untreated cells) were sorted into full growth media. Gating was reestablished after each 1 mL to account for increased uptake of the dye over the course of the experiment (~6 hours). Finally, collected cells were transferred to a 6-well dish well and gently spun down at 100 x g for 10 min and their genomic DNA was extracted using the Zymo Quick-gDNA Miniprep kit, then subjected to PCR and Illumina sequencing as described. Amplicon sequencing was carried out at the MGH DNA core. A readcount table was prepared from raw sequencing files using MAGeCK version 0.5.9.4, *count* subprogram, using standard parameters with 22 bp 5’ trimming. Readcounts were sorted to reveal genes in the top 1000 of all sgRNAs (minimum of 4 or more sgRNAs).

### Fly stocks and maintenance:

All flies in this study were maintained at room temperature and fed a standard fly diet based on the Bloomington Drosophila Stock Center Standard Cornmeal Medium with malt and without soy flour (unless otherwise noted). Stocks obtained from the Bloomington Drosophila Stock Center and the Vienna Drosophila Resource Center [87] were used in this study (listed in S7 Table). The w-;; *eya composite*-GAL4 line was a gift from Justin Kumar (Indiana University Bloomington) and was characterized previously [88]. The "ER stress model" contains *GMR-GAL4* and *UAS-Rh1^G69D^* on the second chromosome and has been previously described [49–51].

The *DPAGT1* model was generated as follows. The *eya composite-*GAL4 and UAS-*Alg7* RNAi line (BDSC #53264) were crossed to create an *eya composite-*GAL4/UAS-*Alg7* RNAi (III) line. This line was then crossed to a balancer +/TM3, Dfd-YFP, *Sb* (III) and progeny were examined for crossover events. Progeny with small, rough eyes and *Sb* were collected, maintained for stability of the phenotype, and referred to as the *DPAGT1* model (*w-*, *y-*, *v-*, *sc-*, *sev-; +/+*; (*eya composite*-GAL4, w+, P[sc+, y+, v+, *Alg7* RNAi])/TM3, Dfd-YFP, *Sb*).

### Eye imaging and quantification:

Adult female flies (2–7 days old) were collected under CO_2_ anesthesia, then placed on ice and transferred to -80°C for later imaging. Eyes were imaged at 3x magnification (Leica EC3 Camera). Eye area was determined as previously described [50]. All measurements were done blinded to the RNAi line used, and one replicate from each model was measured by a second observer. Qualitative images of all control flies are also included as a reference (S2 and S4 Figs). Note that when two RNAi lines were assayed together, the same control was compared to each. When possible, we used two different RNAi lines to limit the possibility of reagent-specific effects. Complete information on lines used can be found in S7 Table and representative images can be found in S1–S4 Figs. In circumstances where only one RNAi line was available, when possible, multiple genes from the same pathway were tested in order to draw conclusions on the pathway as a whole in order to further limit any reagent-specific effects. For *Dpm1*, the BDSC 50713 RNAi line was lethal in the *DPAGT1* model and its control (S7 Table), and studies with the *Dpm1* BDSC 51396 RNAi line were performed at 18°C in the w-;; *eya* composite-GAL4 control line to increase viability. All *Dpm1* RNAi lines were viable when crossed with the ER stress model.

### Sugar supplementation

To make the glucose- and fructose-supplemented media, our standard media was melted in a microwave and maintained at 95°C on a stir plate. We used this melted media as-is for the control and added glucose or fructose to a final concentration of 100mM to a separate set of vials and stirred until it was fully dissolved to a total volume of ~8.5ml/vial. The sugar adds approximately 0.6 calories/tube (that are ~6 calories per 8.5ml [89], when accounting for our added malt), indicating near-isocaloric levels (within ~10%) between each tube. Flies were mated in these vials and removed after egg laying, allowing progeny to feed on sugar-supplemented media from hatching. Timing and quantitative imaging of these progeny was performed as described above.

### Statistics

Genetic overlap representation factors and probability statistics were calculated via "Nematode bioinformatics" (http://nemates.org). For the CDG gene overlap analysis, we used the DIOPT ortholog finder (https://www.flyrnai.org/cgi-bin/DRSC_orthologs.pl) [26] and analyzed any hits with a DIOPT score of 5 or greater.

Gene Ontology (GO) analysis was performed with the PANTHER Overrepresentation test using the "GO biological process complete" data set, compared to all *Drosophila* genes, and using the default parameters (Fisher’s Exact, False Discovery Rate) [90,91]. We also indicate in S7 Table if individual gene orthologs are associated with other relevant GO terms in *H*. *sapiens* (via http://www.pantherdb.org/).

Eye size comparisons were analyzed using the Student’s t test. When comparing groups of RNAi lines to a single control, Bonferroni multiple testing correction was used. Venn diagram and graphs of eye size comparisons were made using the statistical software R [92].

## Supporting information

S1 Fig Qualitative comparisons of primary GDP-mannose, hexosamine, and glycolysis RNAi lines in *DPAGT1* and ER stress models. Representative images from two replicates of RNAi line crosses to both the *DPAGT1* and ER stress models are shown here. This also includes representative images from the RNAi line-specific controls for each cross. RNAi lines used, including specific stock numbers, are included to the left of each image set. Quantification of these eye crosses are included in S7 Table.
(TIF)

S2 Fig Qualitative comparisons of primary GDP-mannose, hexosamine, and glycolysis RNAi lines in *DPAGT1* and ER stress model control lines. Representative images from two replicates of RNAi line crosses to both the *eya composite-GAL4 and GMR-GAL4* control lines are shown here. This also includes representative images from the RNAi line-specific controls for each cross. RNAi lines used, including specific stock numbers, are included to the left of each image set.
(TIF)

S3 Fig Qualitative comparisons of downstream *Dpm1* glycosylation pathway RNAi lines in *DPAGT1* and ER stress models. Representative images from two replicates of RNAi line crosses to both the *DPAGT1* and ER stress models are shown here. This also includes representative images from the RNAi line-specific controls for each cross. RNAi lines used, including specific stock numbers, are included to the left of each image set. Quantification of these eye crosses is included in S7 Table.
(TIF)

S4 Fig Qualitative comparisons of downstream *Dpm1* glycosylation pathway RNAi lines in *DPAGT1* and ER stress model control lines. Representative images from two replicates of RNAi line crosses to both the *eya composite-GAL4 and GMR-GAL4* control lines are shown here. This also includes representative images from the RNAi line-specific controls for each cross. RNAi lines used, including specific stock numbers, are included to the left of each image set.
(TIF)

S5 Fig Simplified model of glycolysis and TCA cycle with labels for gene knockouts causing resistance or sensitivity. Fly gene names are listed, as these genes are derived from the S2 CRISPR knockout guide RNA library. All resistance and sensitivity metrics are based on lfc values from the survival screen.
(PDF)

S1 Table List of all gene Log-fold change (lfc) data used in analysis and Volcano plot. The first tab includes the raw sgRNA enrichment scores (tunicamycin-vs-untreated) 4–8, as well as the median values of those sgRNAs used in subsequent analyses. The second tab includes the adjusted lfc values and p-values used to create the volcano plot in Fig 1.
(XLSX)

S2 Table Comparison of fly orthologs of human CDG genes to survival screen candidate genes. The first tab includes the full list of human CDGs derived from The Essentials of Glycobiology and Invitae panel, the derived fly orthologs via their DIOPT score, a finalized list removing duplicate orthologs alongside their lfc value from the survival screen, and finally the overrepresentation test analysis performed. The second tab includes human CDGs for which no fly ortholog was found at a DIOPT score of <5.
(XLSX)

S3 Table Gene Ontology (GO) analysis of all survival screen candidate genes. This includes the output of the PANTHER gene list analysis from the combined input of resistance and sensitivity candidate genes at an lfc of ≥0.5 or ≤-0.5 from the survival screen in Fig 1 (with re-labeled columns to improve readability). Categories are sorted by fold enrichment.
(XLSX)

S4 Table List of all candidate genes from the ConA screen. The first tab includes only genes that had at least one sgRNA in the highest ~1% of fluorescence of all sgRNAs in two biological replicates. This includes information on specific guides, and the final list of genes found (FBgn number and Fly gene name). The second tab is the raw data from the ConA experiment, including information on specific guide RNAs and their abundance in either replicate.
(XLSX)

S5 Table Gene Ontology (GO) analysis of all ConA screen candidate genes. This includes the output of the PANTHER gene list analysis from the input of candidate genes that improved ConA phenotypes in Fig 2 (with re-labeled columns to improve readability). Categories are sorted by fold enrichment.
(XLSX)

S6 Table Comparison of candidate genes between the survival and ConA screens. This includes the list of resistance candidate genes from the survival screen (S1 Table), all hits from the ConA screen (S4 Table), and the overlapping genes between them (FBgn number and Fly gene name).
(XLSX)

S7 Table List of all primary candidate gene *in vivo* RNAi results. Quantitative results of all RNAi cross replicates to both the *DPAGT1* and ER stress model are listed here. This table includes information on the *Drosophila* gene (name and FBgn number), closest human ortholog, the reasoning on why the gene was tested (GO category, validation, relation to *Dpm1*, etc.), if the gene was a hit in one or both screens, the specific RNAi stock used in each cross, as well as the related pathway function. % change in eye size, confidence interval (derived from the standard deviation of change and listed N), as well as the Student’s t test p-value are indicated for each replicate.
(XLSX)

S8 Table Data on sugar feeding assays. This lists the data on two replicates of the sugar feeding experiments on the *eya composite-GAL4 and GMR-GAL4* control lines and *DPAGT1* and ER stress models. % change in eye size, confidence interval (derived from the standard deviation of change and listed N), as well as the Student’s t test p-value are indicated for each replicate.
(XLSX)
