# Supplementary figures and images for: A genome-wide CRISPR screen identifies DPM1 as a modifier of DPAGT1 deficiency and ER stress
Source: PLoS Genet. 2022 Sep 27;18(9):e1010430. doi: 10.1371/journal.pgen.1010430 (PMC9543880; doi:10.1371/journal.pgen.1010430)

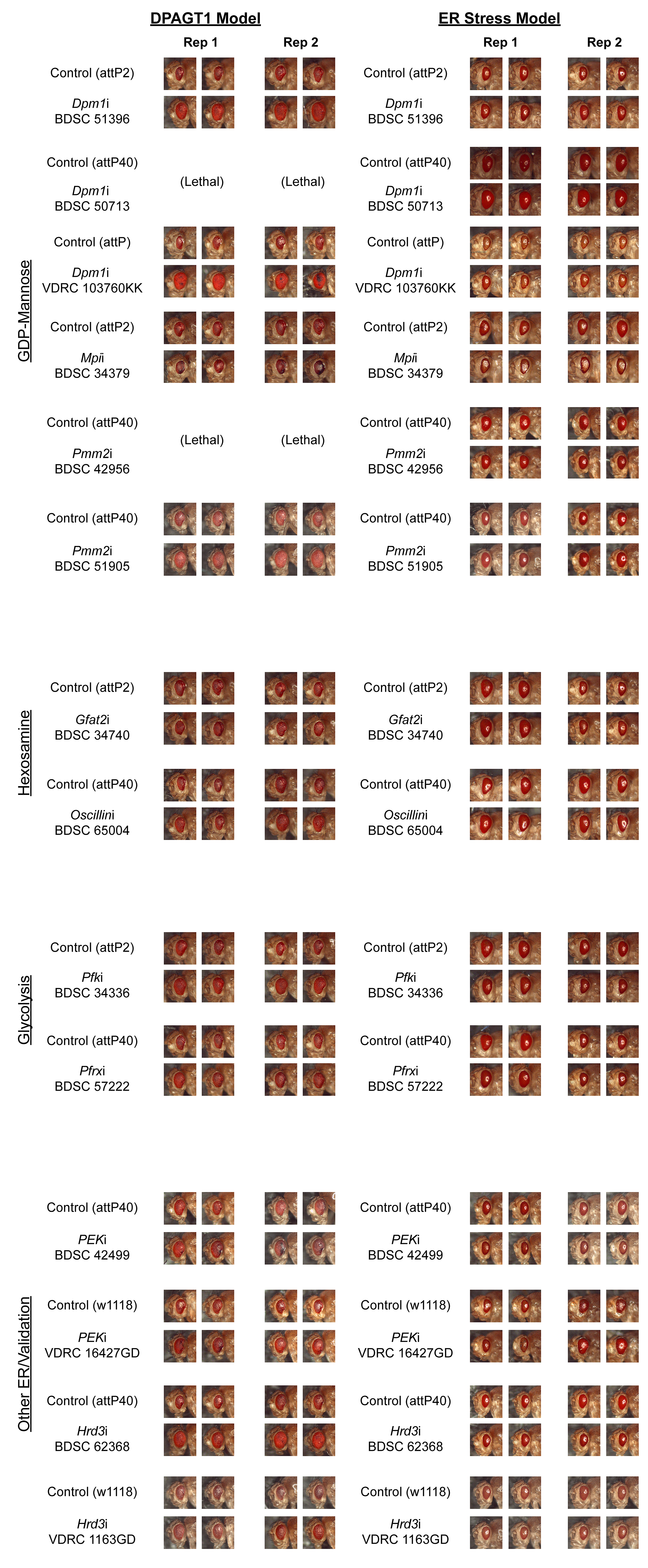

Supplement: S1 Fig — Representative images from two replicates of RNAi line crosses to both the DPAGT1 and ER stress models are shown here. This also includes representative images from the RNAi line-specific controls for each cross. RNAi lines used, including specific stock numbers, are included to the left of each image set. Quantification of these eye crosses are included in S7 Table. (TIF) [file pgen.1010430.s001.tif]

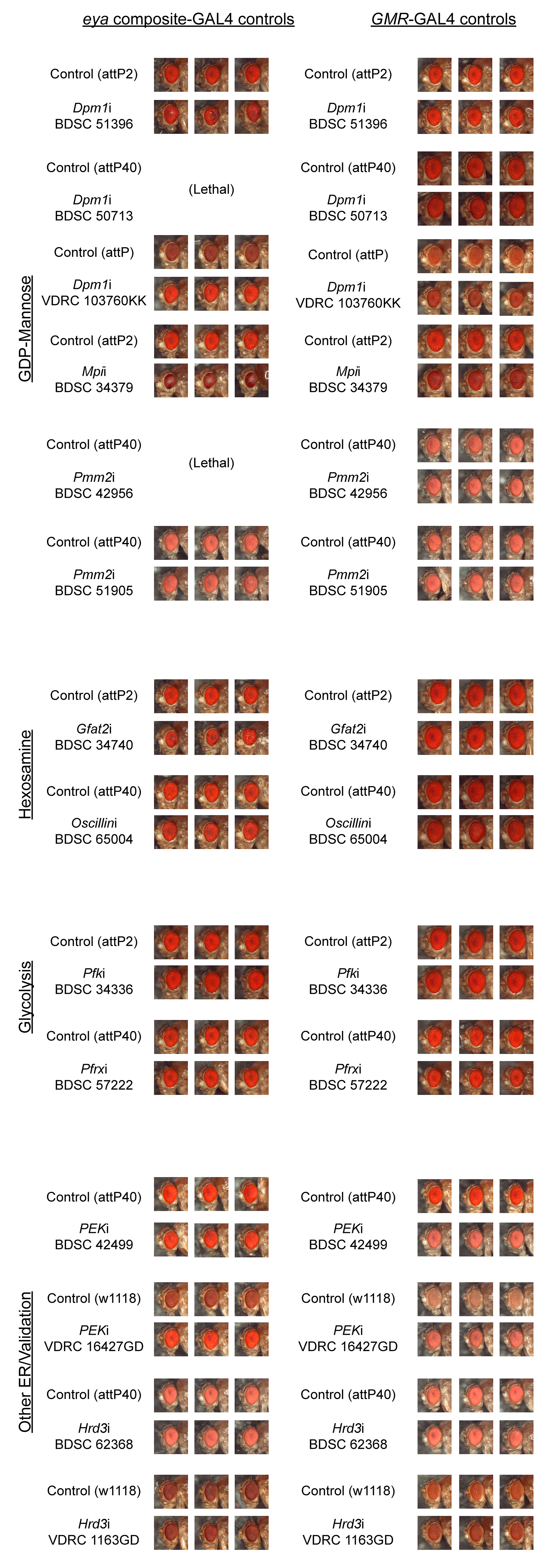

Supplement: S2 Fig — Representative images from two replicates of RNAi line crosses to both the eya composite-GAL4 and GMR-GAL4 control lines are shown here. This also includes representative images from the RNAi line-specific controls for each cross. RNAi lines used, including specific stock numbers, are included to the left of each image set. (TIF) [file pgen.1010430.s002.tif]

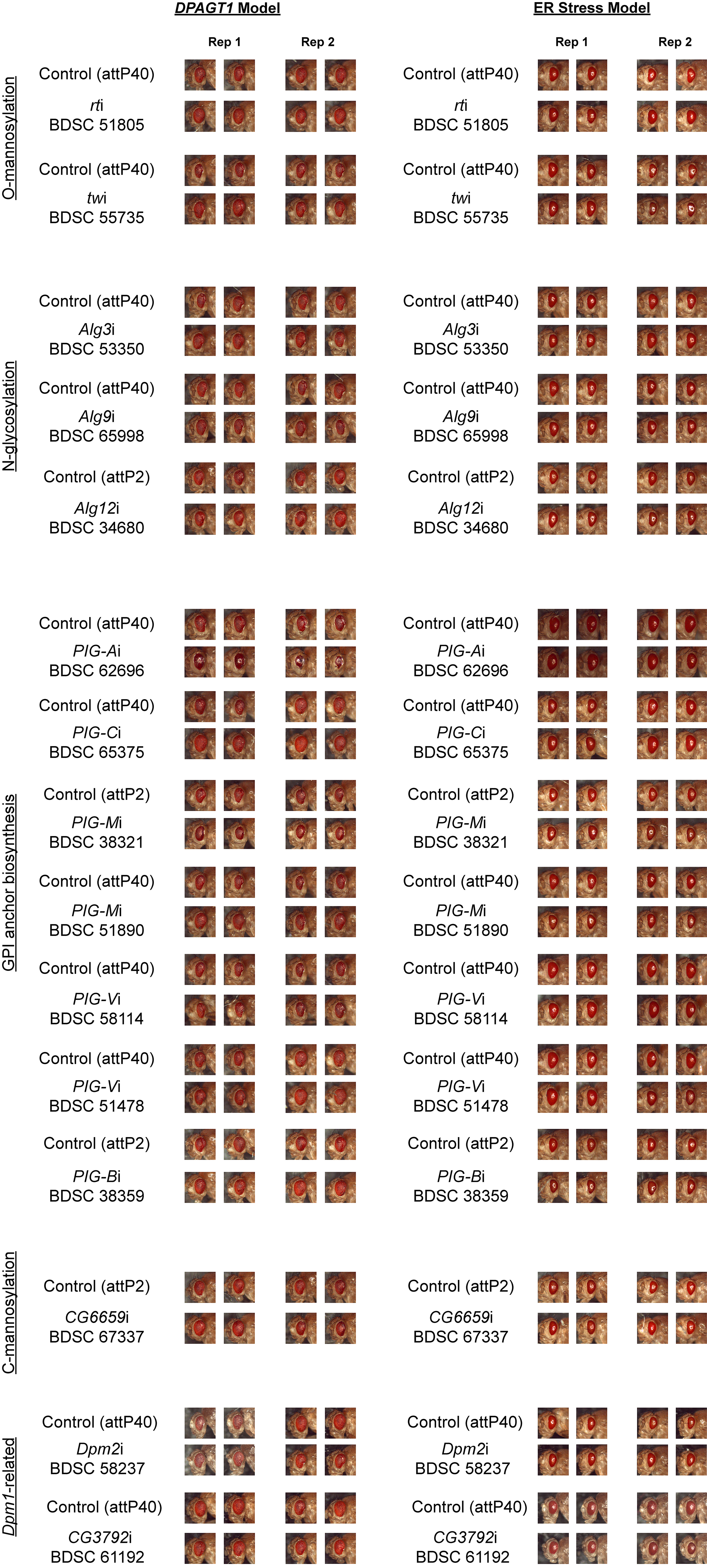

Supplement: S3 Fig — Representative images from two replicates of RNAi line crosses to both the DPAGT1 and ER stress models are shown here. This also includes representative images from the RNAi line-specific controls for each cross. RNAi lines used, including specific stock numbers, are included to the left of each image set. Quantification of these eye crosses is included in S7 Table. (TIF) [file pgen.1010430.s003.tif]

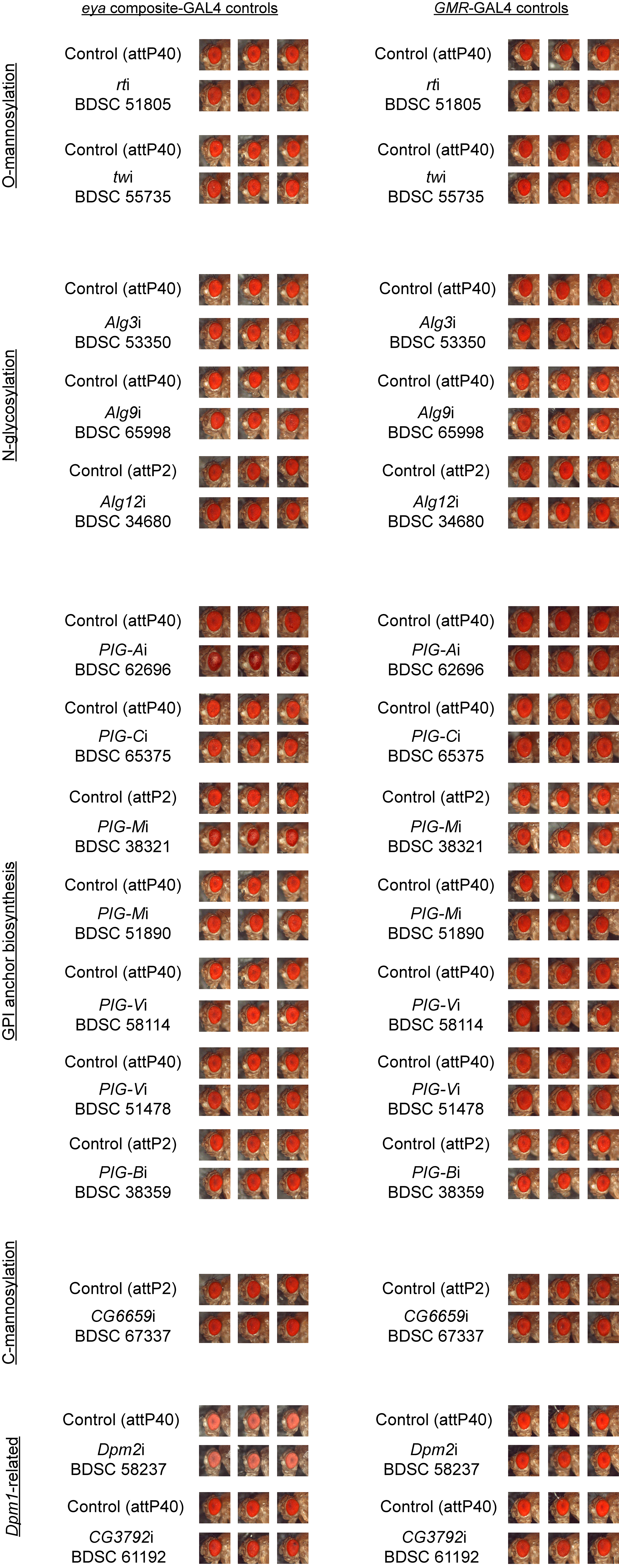

Supplement: S4 Fig — Representative images from two replicates of RNAi line crosses to both the eya composite-GAL4 and GMR-GAL4 control lines are shown here. This also includes representative images from the RNAi line-specific controls for each cross. RNAi lines used, including specific stock numbers, are included to the left of each image set. (TIF) [file pgen.1010430.s004.tif]

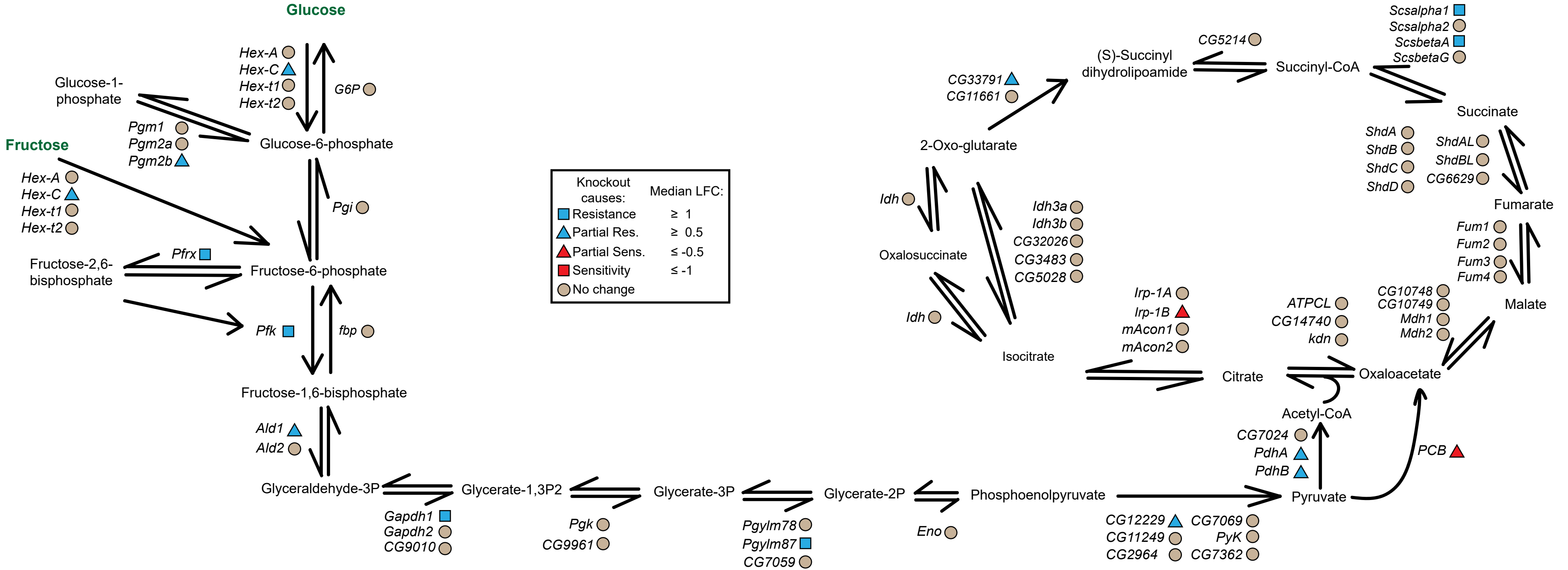

Supplement: S5 Fig — Fly gene names are listed, as these genes are derived from the S2 CRISPR knockout guide RNA library. All resistance and sensitivity metrics are based on lfc values from the survival screen. (PDF) [file pgen.1010430.s005.pdf]
